# Formation of Self-Healing Organic Coatings for Corrosion Protection of Al Alloys by Dispersion of Spherical and Fibrous Capsules

**DOI:** 10.3390/ma16083018

**Published:** 2023-04-11

**Authors:** Makoto Chiba, Yuki Tsuji, Rin Takada, Yuri Eguchi, Hideaki Takahashi

**Affiliations:** National Institute of Technology, Asahikawa College, Asahikawa 071-8142, Japan; mchiba.lab.asnct@gmail.com (Y.T.); c184124@edu.asahikawa-nct.ac.jp (R.T.); c194104@edu.asahikawa-nct.ac.jp (Y.E.); htsapp@mba.ocn.ne.jp (H.T.)

**Keywords:** corrosion protection, self-healing coating, spherical and fibrous capsules

## Abstract

In previous works, we developed a self-healing organic coating with dispersed spherical capsules for corrosion protection. The capsule consisted of a polyurethane shell and healing agent as the inner. When the coating was damaged physically, the capsules were broken, and the healing agent was released from the broken capsules to the damaged area. The healing agent could react with moisture in the air to form the self-healing structure and cover the damaged area of coating. In the present investigation, a self-healing organic coating with spherical and fibrous capsules was formed on aluminum alloys. The corrosion behavior of the specimen coated with the self-healing coating was examined in a Cu^2+^/Cl^−^ solution after physical damage, and it was found that no corrosion occurred during the corrosion test. This is discussed in terms of the high healing ability of fibrous capsules as a result of the high projected area.

## 1. Introduction

**Figure 1:**
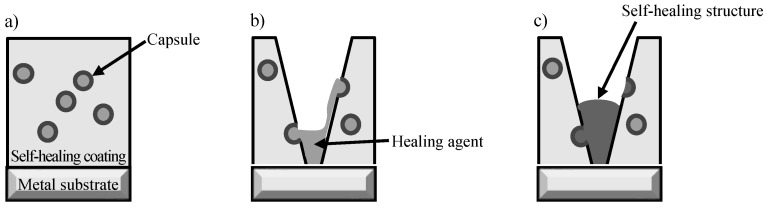
Schematic illustration of self-healing coating with dispersed capsules containing a healing agent. (**a**) Self-healing coating after aging. (**b**) Damage to coating caused by a cutter blade and the flowing out of the healing agent. (**c**) Formation of the self-healing structure through the reaction of the healing agent with moisture in the air.

Metallic materials are widely used for buildings and infrastructure, but they experience corrosion, leading to a degradation in their performance and safety. The cost of losses due to corrosion and corrosion protection is high throughout the world. These values have been reported to be almost 1–5% of the GNP or GDP of many countries [1,2,3,4,5]. One of the strategies for the improvement of corrosion protection in these materials and for the extension of the lifetime of products is the formation of protective layers on the surface [6,7,8,9,10]. In particular, the formation of an organic coating is one of most popular and important techniques and a considerable amount is spent on it for corrosion protection by all countries [11,12,13,14]. However, the high corrosion protection of the metal substrate through the formation of organic coating layers may be easily lost when the coating is locally broken by mechanical damage. Local corrosion, including pitting corrosion and filiform corrosion, among others, occurs due to the exposure of the metal substrate to the corrosive surroundings at the damaged area of the coating. Recently, in order to compensate for the weak aspects of corrosion protection through the formation of organic coatings, self-healing coating for corrosion protection of the metal substrate is attractive as a maintenance-free treatment. Several kinds of coatings with self-healing abilities have been proposed [15,16,17,18,19,20,21,22,23,24,25]. Here, the authors focus on a self-healing coating with dispersed capsules containing a healing agent for the coating [26,27]. This coating can achieve self-healing through the mechanism shown in Figure 1. Figure 1a shows a corrosion-protective organic coating with dispersed capsules containing diisocyanate as a healing agent for the coating, on a metal substrate. When this coating is damaged physically, some capsules dispersed in the coating break and the healing agent flows out from the broken capsule to the damaged area of the coating, as shown in Figure 1b. The healing agent reacts with moisture in the air (Equations (1) and (2)) to form a self-healing structure, polyurea, as shown in Figure 1c.

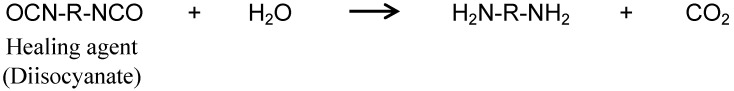
(1)

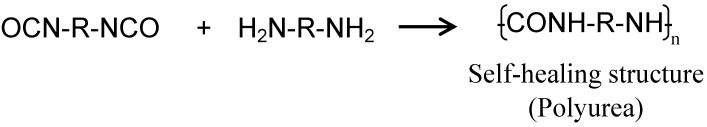
(2)

The self-healing structure covers the exposed area of the metal substrate and the corrosion protection of the metal substrate is maintained at a high level after damage to the coated layer.

**Figure 2:**
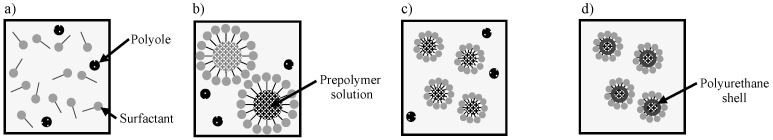
Schematic illustration of the formation of spherical capsules containing healing agent. (**a**) Polyol and surfactant solution. (**b**) Formation of prepolymer micelles by dripping prepolymer solution into (**a**). (**c**) Formation of small prepolymer micelles by vigorous agitation. (**d**) Formation of spherical capsules consisting of polyurethane shell and self-healing agent content.

According to the self-healing mechanism of the coating, the self-healing ability of this coating heavily depends on the shape, size, and structure of the capsules dispersed in the coating. The capsule containing a healing agent can be synthesized by the process shown in Figure 2. First, the organic solvent solution of the prepolymer is synthesized by reacting diisocyanate with polyol, as shown in Equation (3).

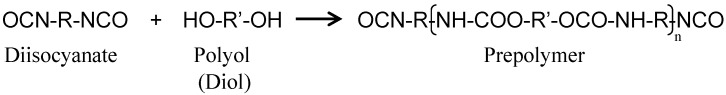
(3)

The synthesized prepolymer solution is dripped to the polyol and surfactant solution. As the prepolymer solution hardly dissolves in the polyol and surfactant solution, the droplets of prepolymer solution with a spherical shape are dispersed in the polyol and surfactant solution, as shown in Figure 2a. When this mixture is agitated vigorously, the droplets of the prepolymer become smaller (Figure 2b) and the surfactant is adsorbed on the surface of the micro-droplets, forming an emulsion (Figure 2c). Furthermore, the reaction shown in Equation (4) occurs only at the interface between the oil phase and water phase to form spherical polyurethane capsules containing the healing agent, diisocyanate (Figure 2d). In a previous study [26,27], the self-healing coating produced by the above procedure, however, did not show a high performance for corrosion protection because of the formation of only small amounts of the healing structure after being damaged. Therefore, it is necessary to improve the self-healing ability of this coating by dispersing capsules with different shapes.

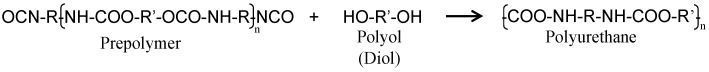
(4)

Considering the self-healing mechanism of the coating, the healing ability of the coating heavily depends on the amount of healing agent that flows out from the broken capsules to the damaged area, so that this is related to the amount of healing agent contained in the capsules. The amount of healing agent in the capsules can be changed by changing shape of the capsules.

**Figure 3:**
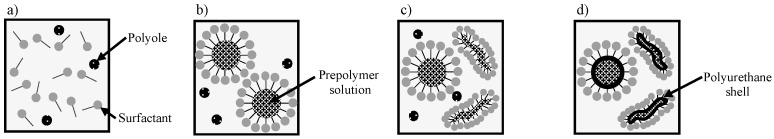
Schematic illustration of the formation of mixed capsules with spherical and fibrous shape, containing the healing agent. (**a**) Polyol and surfactant solution. (**b**) Formation of prepolymer micelles by dripping prepolymer solution with high viscosity into (**a**). (**c**) Formation of spherical and fibrous micelles by agitation at a low speed. (**d**) Formation of spherical and fibrous capsules consisting of polyurethane shell and self-healing agent content.

The shape of the capsules can be controlled by the condition of the capsule synthesis. In a previous study [27], mixed capsules with spherical and fibrous shapes were obtained using concentrated prepolymer solutions. First, the prepolymer solution was heated in order to concentrate the solution (Figure 3a), and then the concentrated prepolymer solution was dripped into the polyol and surfactant solution with a low agitation speed such as 300 rpm (Figure 3b). During dripping, some of the prepolymer solution formed small spherical drops in the polyol and surfactant solution, and the rest became fibrous drops (Figure 3c). This is because the viscosity of the concentrated polymer solution was high enough to form fibrous drops at a low agitation speed. Thus, mixed capsules with a spherical and fibrous shape were obtained by dripping the prepolymer solution with a high concentration into the polyol and surfactant solution under agitation of 300 rpm (Figure 3d). However, the ratio of fibrous capsules to all capsules was less than 10% and there was only a slight difference in self-healing ability between the coating dispersed with spherical/fibrous capsules and that with spherical capsules. The self-healing ability of the coating become higher as the ratio of fibrous capsules increased.

The purpose of this investigation was to examine the effect of the shape of capsules dispersed in an organic coating on the progress of corrosion after physical damaging. In the present investigation, corrosion protection through the formation of self-healing coating with dispersed capsules on Al alloys was focused on. This is because Al and its alloys are often used for automobile, aerospace, and so on [28,29], due to their excellent properties, such as strength, lightness, cost, processability, and recyclability. Three kinds of organic coatings were formed on Al alloys: polyurethane coating without capsules, with dispersed spherical capsules, and with dispersed spherical and fibrous capsules. The corrosion behavior of the specimens covered with the coatings was compared using corrosion tests in the Cu^2+^/Cl^−^ solution after physical damage from a cutter blade.

## 2. Experimental

### 2.1. Synthesis of Capsules Containing Healing Agent

**Figure 4:**
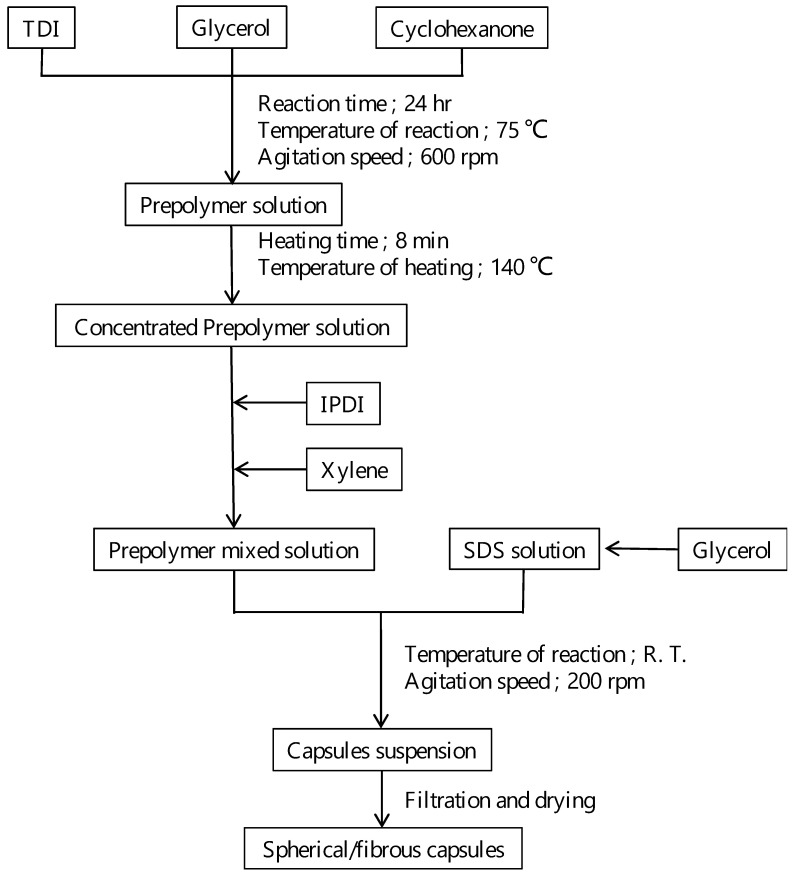
Flow diagram of the formation of spherical and fibrous capsules containing the healing agent, IPDI.

The procedure of the synthesis of mixed capsules with fibrous and spherical shape is summarized in Figure 4 [26,27]. The prepolymer solution was synthesized by the reactions between tolylene-2,4-diisocyanate (TDI) and glycerol in cyclohexanone as a solvent, at 75 °C with 600 rpm agitation for 24 h. This reaction is shown in Equation (5).

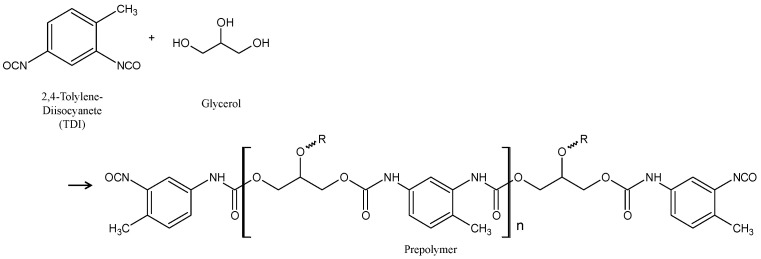
(5)

This solution was heated at 140 °C for 8 min. At this temperature, the cyclohexanone evaporated, but prepolymer did not evaporate. Thus, the concentration of prepolymer increased and the viscosity of the prepolymer solution also increased. Then, isophorone diisocyanate (IPDI), as the healing agent of the coating, and xylene, as the solvent, were mixed to the prepolymer solution. The mass ratios in this solution were prepolymer:IPDI:cyclohexanone:xylene = 0.4:0.2:0.1:0.3. Next, this solution was dripped to 50 mL of 3 wt%-sodium dodecyl sulfate (SDS) solution added with 0.5 g-glycerol under agitation of 200 rpm. As described in Section 1, the prepolymer solution formed micelles with spherical and fibrous shapes, as the prepolymer solution did not dissolve in the SDS/glycerol solution. The prepolymer reacted with glycerol, as shown in Equation (6), at the interface between the prepolymer solution and SDS/glycerol solution, to form the shell of the capsule [26,27,30,31].

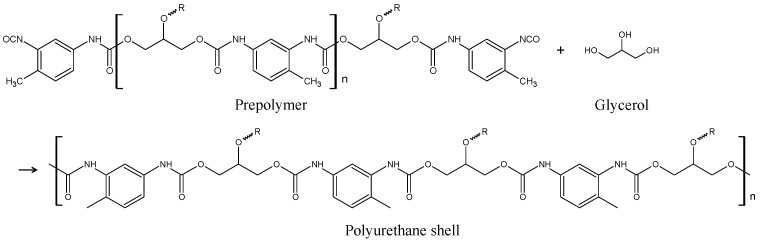
(6)

The size and shape of the synthesized capsules were examined by scanning electron microscopy (SEM) after filtration and drying of the capsules. For comparison, the capsules were synthesized from the prepolymer solution without heating. The mass ratios in this solution were prepolymer:IPDI:cyclohexanone:xylene = 0.13:0.2:0.2:0.47.

### 2.2. Coating

AA1050-Al alloy (purity: > 99.50%, alloy elements: Si, Fe, Cu, etc.) polished mechanically was used as a specimen. The self-healing coating was formed by the following procedure: 0.75 g of the prepolymer solution, 0.1 g of ethylene glycol, and 10 mg of capsules synthesized were mixed. This mixture was coated on the pretreated specimen and aged for 48 h to form a self-healing coating with about 30 µm of the thickness (self-healing coating). The self-heating coating was classified into the self-heating coating with spherical capsules (SHC-SC) and the self-healing coating with spherical/fibrous capsules (SHC-SFC). To compare the effect of the capsules on the corrosion protection, a 30 µm-thick organic coating without capsules was also coated on the specimen (normal coating (NC)). Optical observation of the surface of SHC-SC and SHC-SFC showed a uniform dispersion of capsules without agglomeration. As the capsules were mixed with a prepolymer/ethylene glycol mixture and then coated on the specimen surface, it was assumed that the mixture adhered to the capsule surface before coating. Thus, small semi-spherical mounds of the coating could possibly be formed on capsules larger than 30 μm.

### 2.3. Corrosion Test after Damaging

The specimen with NC, SHC-SC, and SHC-SFC was damaged by the cutter blade with 3 N of load and was aged for 24 h. In order to evaluate the self-healing ability of these coatings, the damaged surface was examined by the secondary electron image from the scanning electron microscopy (SEM; JEOL, JSM-6510LA). Before observation, the specimen was covered with a thin layer of Au using an ion sputter coater. The corrosion protection of the damaged Al alloy specimens covered with NC, SHC-SC, and SHC-SFC was evaluated by a corrosion test in 1.57 mM−CuSO_4_/0.57 M−KCl solution for 24 h at room temperature. Details of the corrosion test were described in a previous study [32]. After this test, the specimen was immersed in a commercially available coating remover and a 10 mass%−phosphoric acid/4 mass%−chromic acid solution, in order to remove the organic coating, corrosion products, and Cu particles deposited during the corrosion test. Then, the trail of corrosion produced on the specimen surface was examined using SEM.

## 3. Results

### 3.1. Shape of Capsules Synthesized from Prepolymer Solution after Heating

**Figure 5:**
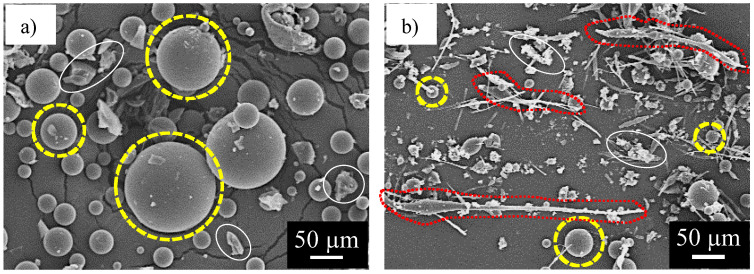
SEM image of the capsules synthesized from the prepolymer solution with IPDI and xylene: (**a**) without heating and (**b**) with heating.

Figure 5 shows SEM images of the capsules synthesized from the prepolymer solution (a) without and (b) with heating for 8 min at 140 °C. There are many spherical capsules with diameters of 10–150 µm, and irregular shaped debris with 10–30 µm in size in Figure 5a. Some capsules are indicated by yellow broken circles, and the debris is indicated by white elliptical circles. The shape of all of the capsules is only spherical. In contrast with this, Figure 5b shows not only spherical capsules with 10–30 µm diameters, but also fibrous capsules with 150–400 µm length and 5–10 µm width. Some of the spherical capsules are indicated by yellow broken circles and some of the fibrous capsules are indicated by red broken elliptical circles.

**Figure 6:**
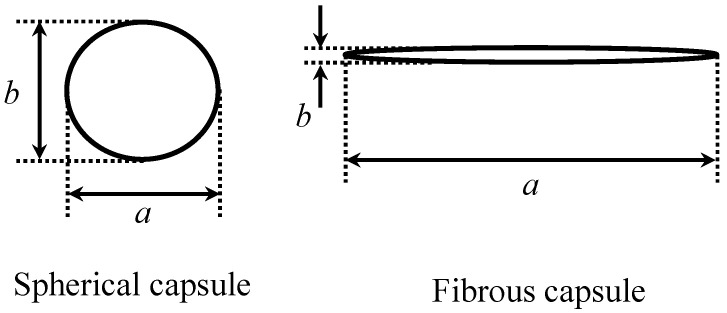
Schematic illustration of the capsule shape projected and the definition of “*a*” and “*b*”.

The shape of these capsules can be quantified using a parameter of flattening, *f*. This can be defined by Equation (7), where the major axis of the projected capsule shape is “*a*” and the minor axis is “*b*”, as shown in Figure 6.

(7) $$f=1-\frac{b}{a}$$

**Figure 7:**
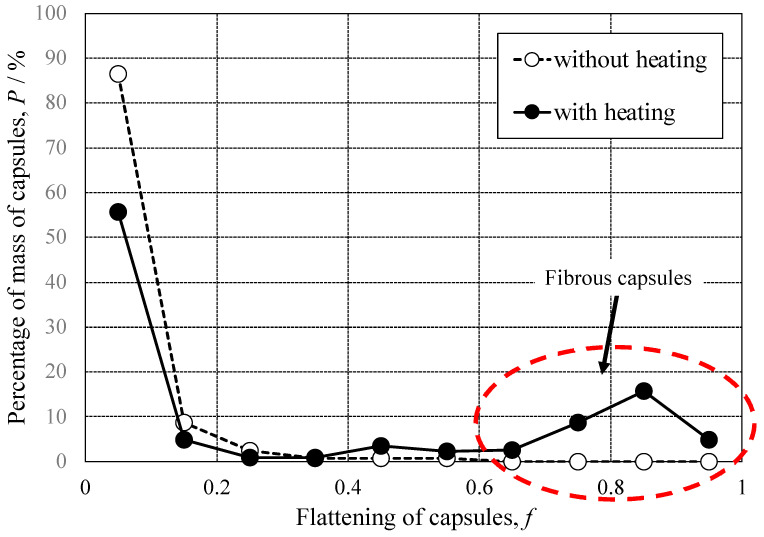
Flattening factor distribution of the capsules synthesized from the prepolymer without and with heating.

From this equation, the value of flattening, *f*, can be calculated as “0”, when the value of “*a*” is the same as that of “*b*”, namely in the case of the projected capsule shape being a perfect circle. On the other hand, if the value of “*a*” is significantly larger than that of “*b*”, the value of *f* is close to “1”. Figure 7 shows the distribution of values of flattening of capsules synthesized from unheated or heated prepolymer solutions. The flattening distribution of capsules synthesized from unheated prepolymers (open circle) shows that most capsules have less than 0.2 for their flattening values and those without a capsule have *f* > 0.5. Thus, the projected shapes of most of the capsules are close to a perfect circle, and no fibrous capsule is synthesized from the prepolymer solution without heating. The flattening distribution of the capsules synthesized from the prepolymer solution with heating (solid circle) shows that the ratio of capsules having less than *f* = 0.2 to all of the capsules occupies 60% and capsules with a *f*-value larger than 0.6 occupy approximately 30%. Here, the capsules with a *f*-value larger than 0.6 are defined as the fibrous ones. Therefore, 70% of the projected shapes for all the capsules were close to a circular shape and 30% were of a fibrous shape when the capsule was synthesized from the prepolymer solution after heating. Conclusively, the ratio of fibrous capsules to all of the capsules increased when synthesizing from a concentrated prepolymer solution under a mild agitation condition.

### 3.2. Self-Healing Ability and Corrosion Protection of Coating Dispersed with Capsules with Spherical and Fibrous Shapes

**Figure 8:**
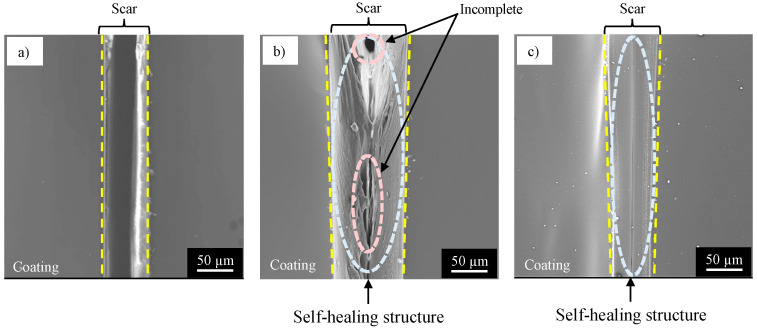
SEM images of the damaged specimen surface covered with a coating (**a**) with no capsule (NC), (**b**) with spherical capsules (SHC-SC), and (**c**) with spherical and fibrous capsules (SHC-SFC).

Figure 8 shows the SEM images of the surface of the damaged specimen covered with (a) NC, (b) SHC-SC, and (c) SHC-SFC. On the specimen with NC, the flat surface of the coating and a sharp scar with 50–70 µm width are observed in Figure 8a. The center of the scar is dark, presuming a deep scar in the coating. The SHC-SC specimen (Figure 8b) shows a scar with 60–80 µm width, and a shallower scar (indicated by elliptic white broken circle) than for the NC specimen. There are narrow black channels (indicated by elliptic, light-blue broken circles) in the bottom of the scar, suggesting that the scar is repaired locally by its self-healing structure. The healing agent reacts with moisture in the air to form a self-healing structure, polyurea, at the damaged area, as shown in Equations (8) and (9).

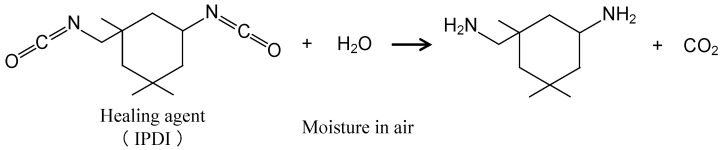
(8)

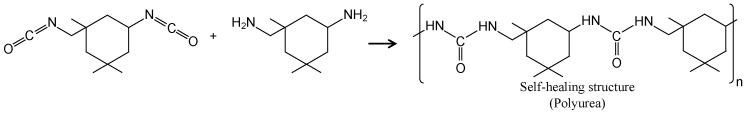
(9)

In Figure 8c, there is a scar with a 50–70 µm width and the scar is shallow. Narrow black channels did not appear, unlike in Figure 8b. This strongly suggests that SHC-SFC can be repaired with a self-healing structure in the entire damaged area.

In the next section, the corrosion protection behavior of the specimens with NC, SHC-SC, and SHC-SFC is shown after damage. The evaluation was carried out by observing the corrosion morphology after the corrosion test in he Cu^2+^/Cl^−^ mixed solution for 24 h at room temperature. In this solution, the corrosion of aluminum occurs by the following reactions [32].

2Al + 3Cu^2+^ → 2Al^3+^ + 3Cu↓ (10)

Al + 3H_2_O → Al(OH)_3_ + (3/2)H_2_ (11)

**Figure 9:**
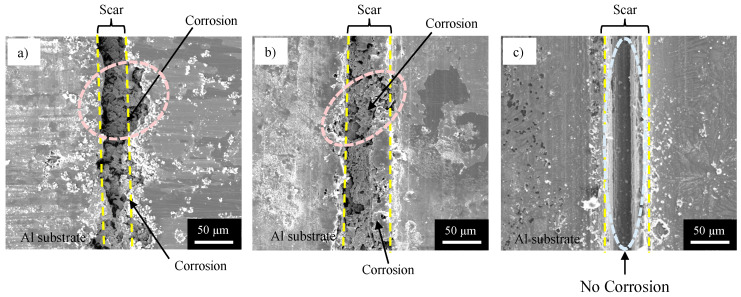
SEM images of the surface of specimens after corrosion test and immersion in coating remover and phosphoric acid/chromic acid solution, obtained for (**a**) NC, (**b**) SHC-SC, and (**c**) SHC-SFC.

From these equations, the corrosion of Al causes the deposition of Cu particles (Equation (10)) and the formation of aluminum hydroxides (Equation (11)). Cu particles and Al hydroxides, as well as the organic coating, can be removed by immersion in a commercially available coating remover and a phosphoric acid/chromic acid solution. Figure 9 shows SEM images of the specimens after physical damage, corrosion test, and immersion in the coating remover and the phosphoric acid/chromic acid solution, obtained for (a) NC, (b) SHC-SC, and (c) SHC-SFC. Figure 9a shows the flat surface of the substrate and a scar with 60–80 µm in width at the center of image. In and around the scar, a semi-spherical pit, indicated by a light-red broken circle is also observed. The bottom of the scar has a rough surface. It can be seen from these results that the scar formed with the cutter blade formed a cavity in the substrate, and that heavy corrosion of the substrate proceeded, forming pits during the corrosion tests.

Figure 9b shows the flat surface of the substrate and a scar with rough surface at the bottom. Semi-spherical pits, indicated by a light-red broken circle, are also observed in and around the scar. However, the size of the pit observed on the specimen with the SHC-SC (Figure 9b) was much smaller than that with NC (Figure 9a). In contrast with these, the specimen with SHC-SFC showed a flat surface of the substrate and a scar with a smooth surface at the bottom, indicated by the light-blue broken line (Figure 9c), and no pit was observed, suggesting no corrosion during the corrosion test. This is because the scar was repaired thoroughly by the healing agent before the corrosion test.

## 4. Discussion

As shown in Figure 9, the SHC-SFC specimen had a much higher corrosion protection than the NC and SHC-SC specimens after physical damage. This was expected because the scar formed with the cutter blade in SHC-SFC was repaired thoroughly by a self-healing structure, but the others were not repaired or were repaired locally (see Figure 8). It is obvious that organic coating (NC) cannot be repaired after physical damaging because there are no capsules that contain the healing agent. The mechanism of repairing SHC-SC and SHC-SFC is discussed below.

**Figure 10:**
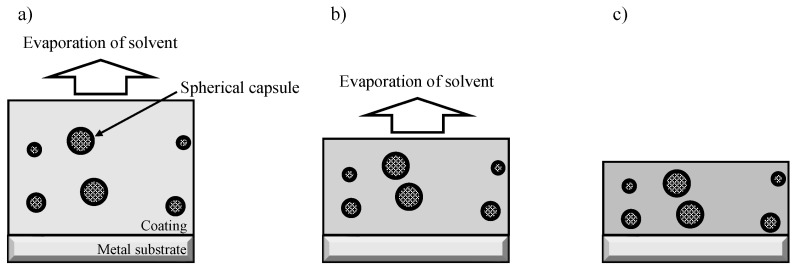
Schematic illustration of the structural changes of the self-healing coating with dispersed spherical capsules (SHC-SC) during aging. (**a**) Just after coating the mixture of prepolymer/ethylene glycol/spherical capsules/cyclohexanone. (**b**) While aging, the reaction of the prepolymer with ethylene glycol and the evaporation of cyclohexanone. (**c**) In the last stage of aging, polyurethane and film thinning formed, which led to an increase in capsule density.

**Figure 11:**
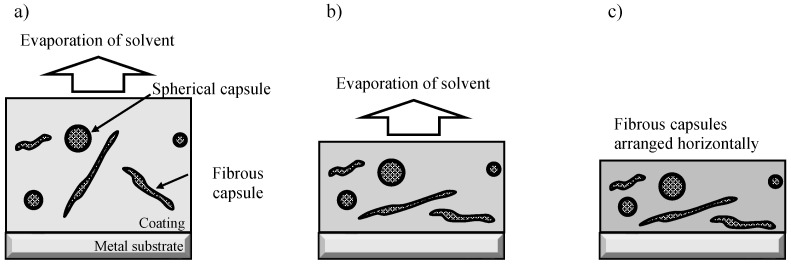
Schematic illustration of the structural change of the self-healing coating with dispersed spherical and fibrous capsules (SHC-SFC) during aging. (**a**) Just after coating the mixture of prepolymer/ethylene glycol/spherical and fibrous capsules/cyclohexanone. (**b**) During aging, reaction of prepolymer with ethylene glycol and evaporation of cyclohexanone. (**c**) In the last stage of aging, the formation of polyurethane and film thinning, leading to an increase in capsule density as well as fibrous capsule being aligned parallel to the specimen surface.

As described in Section 2.2, the total mass of the capsules dispersed in the organic coating was the same in both SHC-SC and SHC-SFC. When the prepolymer/ethylene glycol/capsules/cyclohexanone mixture was coated on the specimen, the prepolymer reacted with ethylene glycol to form polyurethane and the solvent, cyclohexanone, evaporated during aging. Therefore, SHC-SC became thinner, and the capsule density increased slightly during the aging of the coating, as shown in Figure 10. Similarly, in the case of SHC-SFC, the coating became thinner during aging, leading to an increase in capsule density. In addition, the fibrous capsules were expected to be aligned nearly parallel to the specimen surface, as shown in Figure 11.

As described in Section 3.1, the *f* value of the fibrous capsule is approximately 0.6–0.9. Namely, the ratio of the long diameter, *a*, to the short diameter, *b*, can be calculated to be 2.5:1 for *f* = 0.6 and 10:1 for *f* = 0.9. Assuming that the cross-section of the fibrous capsule is a circle, the volume *V_f_* is given by Equation (12) and its projected area *S_f_* is given by Equation (13).

(12) $$V_{f}=\frac{4}{3}\pi \left(\frac{a}{2}\right){\left(\frac{b}{2}\right)}^{2}$$

(13) $$S_{f}=\pi \left(\frac{a}{2}\right)\left(\frac{b}{2}\right)$$

Assuming *b* = 1 and *f* = 0.6, the value of a was calculated to be 2.5, and thus the volume, *V_f,_* of the fibrous capsule was 1.31, and the projected area, *S_f_,* of this capsule was estimated to be 1.96. The diameter of the spherical capsule with the same volume (*V_f_* = 1.31) was calculated to be *a* = 0.68, so that the projected area S_f_ of a spherical capsule was calculated to be 1.45. The projected area of the fibrous capsule was 1.33 times larger than that of the spherical capsule. In the case of *f* = 0.9 and *b* = 1, a was calculated to be 10, and *V_f_* and *S_f_* were 5.23 and 7.85, respectively. The diameter of the spherical capsule with *V_f_* = 5.23 was calculated to be *a* = 2.15, so that the projected area of the spherical capsule was estimated to be *S_f_* = 3.63. Thus, the projected area of the fibrous capsule was 2.16 times larger than that of the spherical capsule. The projected areas of the fibrous capsules were larger than those of the spherical capsules, and the ratio of *S_f_* of fibrous capsules to that of spherical capsules became larger as the *f* value increased.

**Figure 12:**
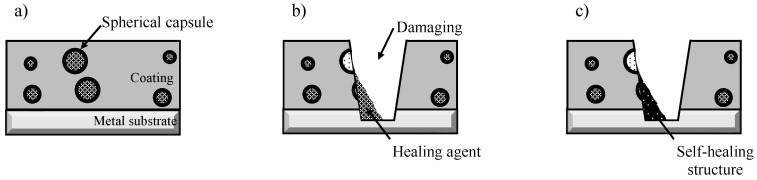
Schematic illustration of the structural change of SHC-SC by physical damaging. (**a**) Just before damaging, polyurethane coating with dispersed spherical capsules. (**b**) Just after damaging, flowing-out of healing agent from broken capsules to damaged area. (**c**) At the last stage of aging, repairing the coating at part of the damaged area with a self-healing structure.

**Figure 13:**
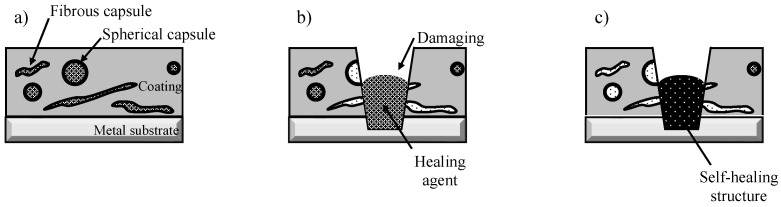
Schematic illustration of the structural change in SHC-SFC by physical damage. (**a**) Just before damage, polyurethane coating with dispersed spherical and fibrous capsules. (**b**) Just after damage, flowing-out of healing agent from broken capsules to damaged area. (**c**) At the last stage of aging, repairing the coating in the whole damaged area with a self-healing structure.

Thus, fibrous capsules aligned parallel to the surface were more likely to be broken than the spherical capsules when the coating was damaged. This is because the projected area of the fibrous capsules was larger than that of the spherical capsules. SHC-SC was repaired partly due to a small amount of healing agent that flowed out from the broken capsules, as shown in Figure 12. In contrast, SHC-SFC was repaired thoroughly due to the large amount of healing agent that flowed out from the broken capsules, as shown in Figure 13.

In previous investigations [26], porous-type anodic oxide films were formed on Al alloys, and nano-pores were filled with a solution containing a corrosion inhibitor before being covered with organic coatings. The specimen was physically damaged with a cutter blade, and corrosion tests were carried out in 1.57 mM–CuSO_4_/0.57 M–KCl solution for 24 h at room temperature. The corrosion protection effect appeared just after physical damage by the inhibitor that flowed out from the nano-pores to the damaged area, but the system could not achieve thorough protection. This might be due to the fast adsorption of the inhibitor on the surface of the damaged area at a low concentration.

Conclusively, the corrosion protection ability of the self-healing coating on the Al alloy substrate heavily depends on systems and the shape of capsules dispersed in the coating. Fibrous capsules were aligned parallel to the surface during aging of the coating, and a large amount of healing agent flowed out from the broken capsules after damage to repair the scar thoroughly.

## 5. Conclusions

In the present investigation, three kinds of organic coatings were formed on aluminum alloys: polyurethane coating without capsules (NC), with dispersed spherical capsules (SHC-SC), and with dispersed spherical and fibrous capsules (SHC-SFC). The corrosion behavior of the coatings was compared by corrosion tests in Cu^2+^/Cl^−^ solution after physical damage with a cutter blade. The following conclusions were drawn.

1. The specimen with NC corroded heavily at the damaged area, suggesting no self-healing.
2. The specimen with SHC-SC corroded partly at the damaged area, suggesting appreciable self-healing due to a small amount of healing agent flowing out from the broken capsules.
3. The specimen with SHC-SFC did not corrode during the corrosion test after damage. This was due to repairing of the scar at the whole damaged area with healing agent, mainly from the fibrous capsules.

## Data Availability

Not applicable.
